# Total femoral replacement for severe femoral bone loss in non-oncologic patients: high complication rates but acceptable functional outcomes

**DOI:** 10.1186/s12891-026-10052-3

**Published:** 2026-06-10

**Authors:** Irene Matellanes Mielgo, Victor Rafael Casas Gállego, Juan B de la Torre Mosquera, Basilio José de la Torre Escuredo

**Affiliations:** 1 Department of Surgery, Medical and Social Sciences, Faculty of Medicine and Health Sciences, Ramón y Cajal Institute of Sanitary Research (IRYCIS), University of Alcala, Alcala de Henares, Madrid, 28034 Spain; 2 2Department of Orthopedic Surgery, University Hospital Ramón y Cajal, Madrid, 28034 Spain

**Keywords:** Total femoral replacement, Megaprosthesis, Limb salvage/preservation, PROMs

## Abstract

**Introduction:**

The management of severe femoral bone loss is a growing challenge in prosthetic revision surgery, especially in patients who have undergone multiple previous surgeries. In these complex scenarios, total femoral replacement (TFR) has been used as a rescue technique to preserve the limb, despite its association with a high rate of complications. Evidence on functional outcomes and patient-reported outcome measures (PROMs) from patients with nononcological indications is limited. The objective of this study was to analyze the clinical and functional results of and the complications associated with TFR in a nononcology series and to identify possible prognostic factors.

**Materials and methods:**

In this retrospective, observational study, 13 consecutive patients who underwent TFR between 2020 and 2024 and who had a minimum follow-up of 18 months were included. Demographic, clinical and surgical variables; implant survival; and postoperative complications were analyzed. Functional evaluations included assessments of the ability to walk, the visual analog scale for pain and collection of PROMs (12-item short form survey (SF-12), Musculoskeletal Tumor Society (MSTS) scale and Western Ontario and McMaster Universities Arthritis Index (WOMAC) test).

**Results:**

The mean patient age was 75.3 years. The main indication for TFR was peri-/interprosthetic fracture (69.2%), followed by aseptic loosening (23.1%). The average number of previous surgeries was 4.8. Implant survival at one year was 100%; however, 61.5% of the patients required at least one reintervention, with periprosthetic infection being the most frequent complication (38.5%). A total of 92.3% of the patients maintained the ability to walk with assistance, and while the PROMs reflected moderate pain control and limited function, they were indicative of basic independence.

**Conclusions:**

Despite the high rate of complications, in patients with severe femoral bone loss, TFR is a valid option for preserving the limb and allowing a faster transition from assisted ambulation in nononcology patients.

## Introduction

Femoral revision surgery is often indicated for managing loss of the remnant femoral bone due to an increase in the number of femoral re-revision procedures—a consequence of the increased patient life expectancy and the complexity of cumulative surgeries [1–3]—as well as in the complications of periprosthetic and interprosthetic fractures with large femoral defects, where the loss is associated with biological complications of fracture consolidation [4, 5]. For all these reasons, clinicians are increasingly faced with cases in which the preservation of the limb is possible only through total femoral replacement (TFR). Thus, the indications for megaprostheses currently extend beyond traditional oncological indications.

Megaprostheses replace not only the femur itself but also the hip and knee joints and can be grouped into two specific classes: tumor-style replacements and intramedular/push-through total femur replacements. The former involves the complete resection of the femoral bone, replacing the entire femur with a prosthesis, leaving no remnant of the patient´s original femur, whereas the latter preserve the patient´s femoral remnant, with the prosthesis inserted through the intramedullary canal, maintaining some of the native femur.

It is a demanding and aggressive procedure with potentially serious complications, but it allows preservation of the limb as well as early mobilization and weight-bearing [4, 6]. The most frequent complication is periprosthetic infections [6–8], occurring in between 25% and 92% of all procedures [4–6, 9].

Moreover, most patients who undergo this procedure are elderly individuals with multiple comorbidities and few functional reserves. Therefore, in this context, patient-reported outcome measures (PROMs) have emerged as essential tools for assessing the most important consequences of the operation: its real functional impact, postoperative quality of life and patient expectations after a surgery of this magnitude. However, studies on the outcomes of revision total hip arthroplasty (rTHA) lack comprehensive data on the relationship between PROMs and TFR. Additionally, studies published to date have consisted of case series that evaluated mid- to long-term outcomes without examining relevant PROMs.

Our objective was to analyze the clinical and functional results in a series of patients with severely compromised functional status, determine the real scope of the procedure, and identify the profiles of patients who, despite the high complexity of the procedure and its associated complications, benefit from this type of surgery and, if necessary, redefine the indications for the procedure. Understanding the factors that predict patient outcomes may help surgeons in discussing clinical expectations with patients undergoing TFR.

## Materials and methods

### Study design

*A retrospective analytical observational study was carried out with data from patients who underwent TFR. The study was approved by the Research Ethics Committee (CEIM registration number: 258/25) and was conducted in accordance with the principles of the Declaration of Helsinki. Patients were informed about the objectives of the study and gave their informed consent to participate.*

### Patients

Patients who underwent TFR between January 2020 and July 2024 were included. A total of 13 procedures in 13 patients were carried out, and the minimum follow-up was 18 months (69 − 18 months). The critical factor in determining whether total femoral replacement is the appropriate reconstruction option is the amount of intact diaphyseal or distal femoral bone remaining for stem fixation; if the size of the remnant bone is less than 120 mm, reconstruction methods involving stem fixation are likely to fail, and total femoral replacement is indicated [4, 11].

The exclusion criteria were age younger than 18 years and TFR performed for oncologic reasons.

Patient data were retrospectively collected via a review of the electronic medical records and postoperative radiographs and included age, sex, body mass index (BMI), diabetes mellitus status, anesthetic risk according to the American Society of Anesthesiologists (ASA) scale, reason for revision surgery (septic-aseptic; peri-/interprosthetic fracture), type of revision (acetabular, femoral, or both), number of previous revision surgeries, and type of femoral defect. Similarly, data on complications such as periprostatic infection, dislocation, and mortality were collected. All the data were collected by one of the authors during the clinical review.

The clinical outcomes evaluated included implant survival and complications associated with the TFR.

### Assessment of femoral defects

The patients’ femoral defects were classified into four types according to the Paprosky classification [10] among which type IIIB and type IV defects are exclusive indicators for TFR as a reconstructive technique to aid in ambulation recovery in elderly patients [11].

### Evaluation of functional outcomes

To evaluate the functional outcomes of the procedure, we analyzed data regarding the ability to walk, as well as the PROMs and postoperative pain using 4 patient-completed questionnaires: the physical and mental component scale of the 12-Item Short Form Health Survey (SF-12) quality of life questionnaire [12]; the Musculoskeletal Tumor Society (MSTS) scale [13], the Western Ontario and McMaster Universities Osteoarthritis Index (WOMAC) [14] and the visual analog scale (VAS) for pain [15]. PROMs were collected at the latest follow-up visit after surgery. Follow-up duration for each patient is reported in Table 1.

**Table 1:** Individual patient data: demographic variables (sex: F = female, M = male), initial cause of arthroplasty and TFR, number of previous surgeries, length of hospitalization, complications, type of TFR, dysmetria and follow-up time

	Sex	ASA	Initial cause of arthroplasty	Cause of TFR	Number of previous surgeries	Complications	Type of TFR	Dysmetria	Follow-up
1	F	II	Gonarthrosis	Aseptic loosening	4	-	Push-through	0.5	5 years and 5 months
2	F	II	Gonarthrosis	Aseptic loosening	6	-	Push-through	0.6	4 years and 6 months
3	F	II	Gonarthrosis	Periprosthetic fracture	5	Acute periprosthetic infection	Push-through	1.5	4 years and 4 months
4	F	II	Gonarthrosis	Interprosthetic fracture	10	Acute periprosthetic infection	Push-through	1	3 years and 6 months
5	F	II	Gonarthrosis	Interprosthetic fracture	3	Acute periprosthetic infection	Tumor-type	9.8	3 years and 7 months
6	F	II	Perisubtrochanteric fracture	Periprosthetic fracture	6	Chronic periprosthetic infection	Push-through	1.4	3 years and 4 months
7	F	II	Gonarthrosis	Periprosthetic fracture	5	Surgical wound dehiscence	Tumor-type	2	2 years and 5 months
8	F	II	Gonarthrosis	Pseudoarthrosis periprosthetic fracture	6	Acute periprosthetic infection	Push-through	1.5	2 years and 7 months
9	M	II	Gonarthrosis	Chronic TKP infection	4	-	Push-through	2	2 years and 8 months
10	F	II	Gonarthrosis	Periprosthetic fracture	4	Common peroneal neuropraxia	Push-through	1.1	1 year and 10 months
11	F	II	Persubtrochanteric fracture	Periprosthetic fracture	1	Surgical wound dehiscence	Push-through	1.3	1 year and 7 months
12	F	II	Gonarthrosis	Aseptic loosening	4	-	Push-through	1.3	1 year and 2 months
13	F	II	Gonarthrosis	Intraoperative periprosthetic fracture	4	Malrotation	Push-through	2	1 year and 3 months

### Implants and surgical technique

**Fig. 1:**
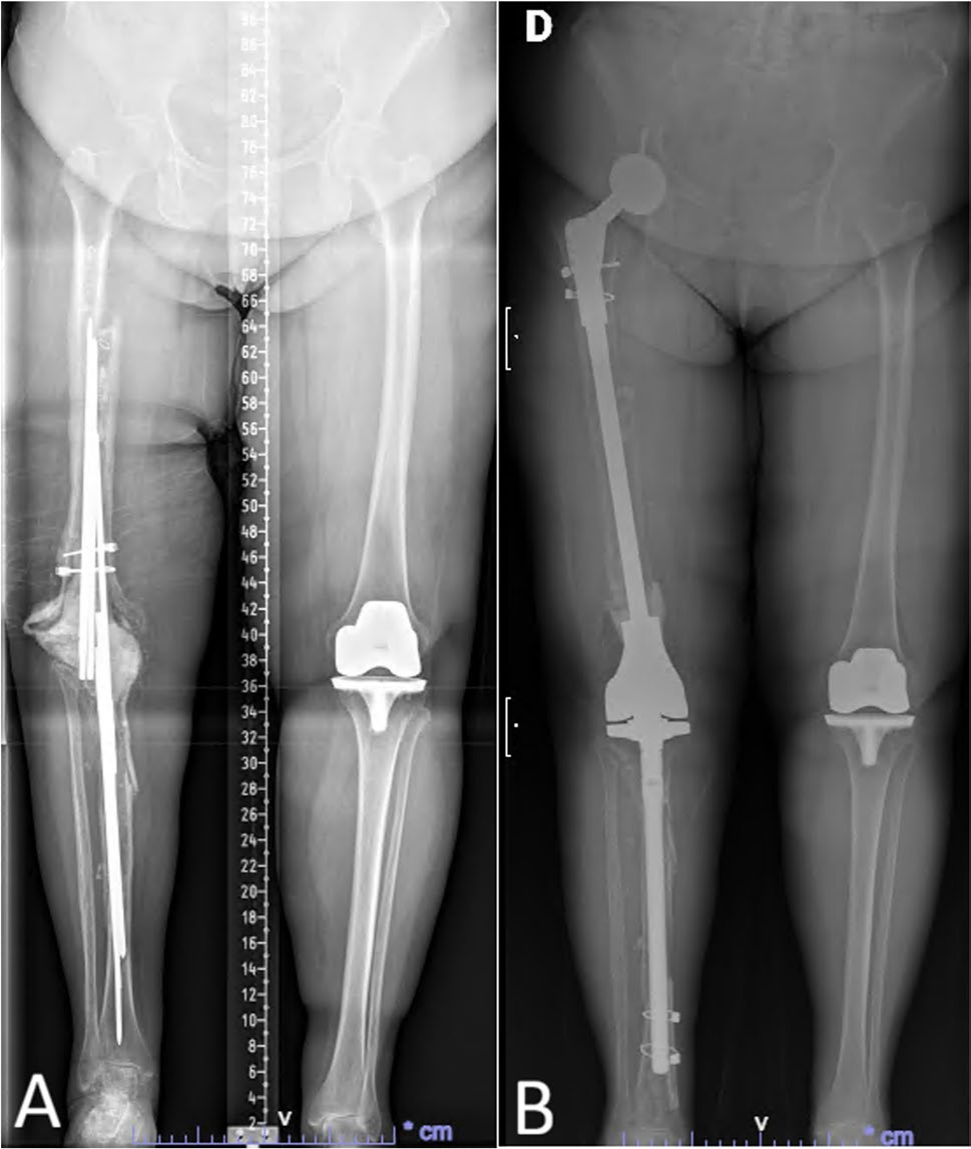
**A** Preoperative anteroposterior radiograph of a woman with knee osteoarthritis complicated by chronic infection, operated on several times, with a fracture of the femur and tibia during the last surgery. **B** Radiograph at three-year follow-up after reconstruction with a total femoral replacement using a push-through stem

**Fig. 2:**
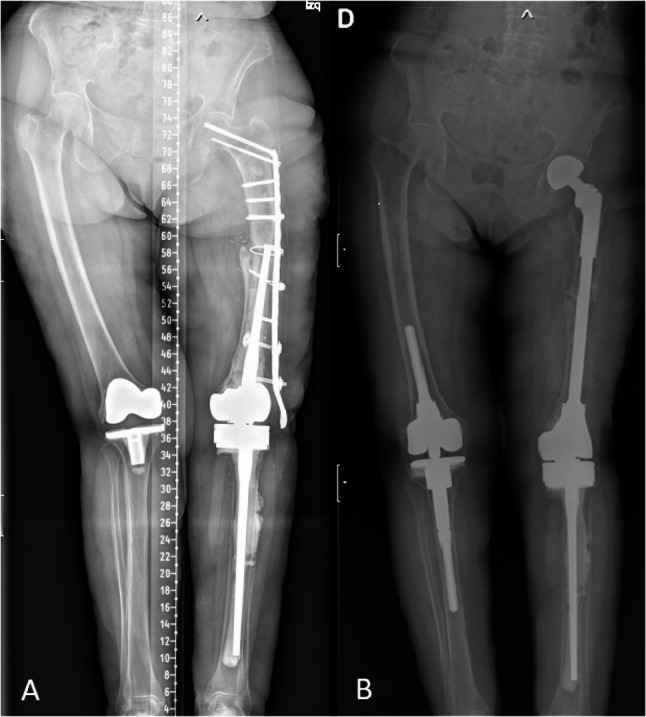
**A** Preoperative anteroposterior radiograph demonstrating failure of fixation of a periprosthetic femoral fracture secondary to femoral nonunion in an elderly female patient. Preoperative cultures were negative. **B** Postoperative radiograph showing one-stage reconstruction with removal of previous hardware and implantation of a total femoral replacement using a push-through fixation system. Intraoperative cultures subsequently yielded Staphylococcus epidermidis

All patients were implanted with the MEGASYSTEM-C system (Waldemar-Link, Hamburg, Germany). This modular revision system allows total femoral reconstruction involving both the hip and knee revision components with an adjustable femoral module and a rotational hinge in the knee. In most cases, an intramedullary (push-through) stem prosthesis was used, while two patients were treated with the tumor-type prosthesis, one because of extensive debridement performed due to periprosthetic infection, and the other because of very little bone remnant after an interprosthetic fracture (Figs. 1 and 2).

In all patients, before TFR was performed, the presence of infection was ruled out following the consensus of the European Bone and Joint Infection Society (EBJIS) 2021 [16].

After the patient was placed in the lateral decubitus position, the procedure began with implantation of the knee prosthesis through a medial parapatellar approach. Once the knee replacement was completed, the hip was replaced using a posterolateral approach. In cases in which an intramedullary (push-through) stem was implanted, both approaches were established without creating a communication between them. If this was not possible or if it was necessary to implant a tumor-type stem, communication was established between the hip and knee approaches. When the greater trochanter was present, to obtain good access for the removal of the previous prosthesis or for the implantation of the intramedullary stem, an extended trochanteric osteotomy was performed. The first cases in the series were not managed with trochanteric osteotomy, as it is associated with intraoperative fractures at the level of the proximal femur.

Once the cemented tibial implant was placed, the femoral component was implanted at the knee, with the intramedullary stem placed in the femoral diaphyseal remnant. It is essential to apply cement to the distal end of the femur to fixate the bone with the prosthesis; solidification of the cement in the distal bone remnant stabilizes the femoral component and prevents bone rotation over the prosthesis, thus contributing to maintaining correct and stable anteversion of the implant. We did not cement the rest of the femoral canal for any of our patients. From the hip approach, we subsequently proceeded to connecting the proximal stem. At this point, component tests were performed to adjust the implant to the appropriate length and achieve suitable stability. Finally, the definitive hip components were implanted, and the procedure was completed by closing the trochanteric osteotomy or reconstructing the remnant of the gluteus medius.

### Statistical analysis

A descriptive analysis of the quantitative variables was performed using measures of central tendency (means) and dispersion (standard deviations), while qualitative variables are expressed as absolute frequencies and percentages. The analysis was performed using STATA MP v17.0 statistical software (StataCorp, College Station, TX, USA).

## Results

### Patients

All our surgical cases involved femoral defects of nononcological origin.

The data of 13 patients (12 women, 1 man), mean age 75.3 years at the time of implantation, were reviewed. The most frequent cause of TFS was peri-/interprosthetic fracture, which was observed in 69.2% (*n* = 9) of the patients, followed by aseptic loosening in 23.1% (*n* = 3); the remaining patient was managed with TFR due to a chronic periprosthetic infection. The majority of patients (84.6%; *n* = 11) had a history of primary total knee arthroplasty, after which, owing to different complications, they developed a distal femoral defect that led to the indication for TFR (Table 1).

On average, the patients had previously undergone 4.8 ± 2.1 (SD) surgeries before undergoing TFR, with an average hospital stay of 31.9 ± 19.4 days. The minimum recorded follow-up was 18 months, and no deaths were documented during the observation period.

### Complications

**Fig. 3:**
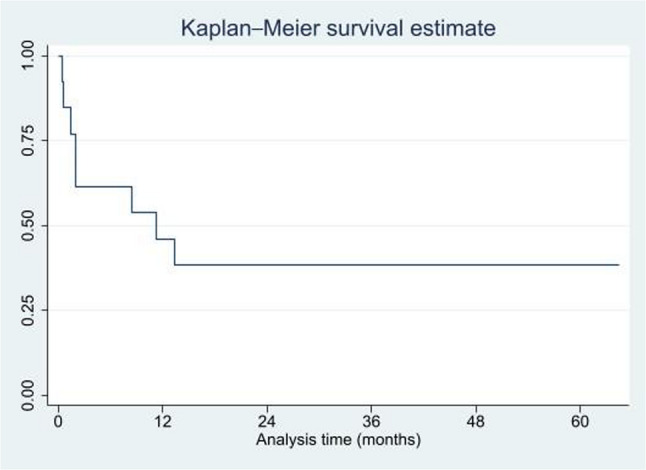
Kaplan‒Meier curve of the complications that required a new surgical intervention. Most reinterventions occurred during the first year of follow-up

The implant survival rate at one year was 100%. However, 61.5% of the patients (*n* = 8) required at least one reintervention because of major complications. The most frequent of these was periprosthetic infection (38.50%, *n* = 5), followed by problems related to surgical wound healing, all of them around the knee (15.40%, *n* = 2). All patients were treated with extensive debridement, polyethylene replacement of the knee prosthesis, implant retention, and antibiotic treatment. In one patient, a new intervention was necessary because of malrotation of the femoral component. The remaining patients (38.50%, *n* = 5) had no complications during follow-up. Kaplan‒Meier analysis (Fig. 3) showed a considerable incidence of early complications as well as a new complication that required surgical intervention at approximately the first postoperative year.

### Functional outcomes

In terms of the final functional results, all the patients in the study except one retained the ability to ambulate. Most of these patients required some type of technical assistance to walk, such as one (30.8%, *n* = 4) or two crutches (23.1%, *n* = 3) or a walker (38.5%, *n* = 5). The remaining patient (7.7%, *n* = 1) was unable to walk; this patient was only able to transfer from bed to chair and required a wheelchair for movement. The data of each patient are shown in Table 2.

**Table 2:** Individual patient data: Ambulation, PROMs and pain scores

	Ambulation	SF-12: PCS score	SF-12: MCS score	MSTS score	WOMAC Pain score	WOMAC Rigidity score	WOMAC Functional capacity score	Numeric pain score
1	2 crutches	24.88	56.51	10	5	4	40	3
2	2 crutches	27.49	46.77	8	5	2	39	3
3	Walker	28.55	25.77	8	6	2	55	1
4	Walker	26.03	53.82	10	5	1	35	3
5	Nonambulatory	31.49	43.91	6	5	3	42	5
6	Walker	24.53	49.01	6	12	6	36	8
7	1 crutch	36.63	44.16	19	4	1	22	2
8	Walker	31.83	38.89	9	5	0	40	3
9	1 crutch	30.71	62.55	14	1	0	30	0
10	2 crutches	21.13	61.94	7	3	3	30	4
11	1 crutch	26.19	62.72	11	6	2	32	2
12	Walker	21.32	61.09	10	10	6	34	8
13	1 crutch	28.91	36.72	11	11	4	44	4

In terms of pain control, as evaluated by the VAS scale, the mean score observed in our series was 3.5 (SD: 2.5). With respect to the patient-reported outcome measures, the mean score on the MSTS was 9.92 (SD: 3.52). In the SF-12 questionnaire, the mean physical component score was 27.67 (SD 4.37), and the mean mental component score was 49.53 (SD: 11.58). Finally, the mean WOMAC was 6.00 (SD: 3.16) in the pain domain, 2.62 (SD: 1.98) in the stiffness domain and 36.85 (SD: 8.07) in the functional capacity domain. Together, these results reflect moderate pain control and limited functionality, although they are compatible with assisted ambulation and an acceptable adaptation to the basic activities of daily living.

When the associations between the indications for TFR and PROMs were analyzed, the results were not significantly different, indicating that the reason for performing TFR does not influence the functional results in terms of either residual pain or the ability to ambulate after surgery. Specifically, the association with the PROMs was assessed with the nonparametric Kruskal‒Wallis test, which yielded a p value > 0.05 for PROMs, while Fisher’s exact test was used to evaluate the association with ambulation, which yielded a p value = 0.47. Similarly, we did not find a significant association between the number of previous surgeries and the functional results as evaluated by PROMs and the final ability to ambulate (Mann‒Whitney U test for PROMs, *p* > 0.05; Fisher’s exact test for ambulation, *p* = 0.35).

In terms of age, older patients had better pain control after surgery (*p* = 0.032). However, there was no significant association between older age and walking ability (Kruskall–Wallis, *p* = 0.07), although the p value was close to the significance threshold, suggesting a possible trend that could be confirmed with a larger sample.

An association was observed between the number of previous surgeries and the rate of complications, such that complications that required reintervention were recorded in 43% of patients with ≤ 4 previous surgeries and in 83% of those with ≥ 5 previous surgeries. Although the difference did not reach statistical significance (Fisher’s exact test, *p* = 0.17)—probably because of the small sample size, patients with a greater number of previous procedures tended to have a higher risk of complications (relative risk (RR) of 1.94 [95% CI: 0.79–4.76]).

**Fig. 4:**
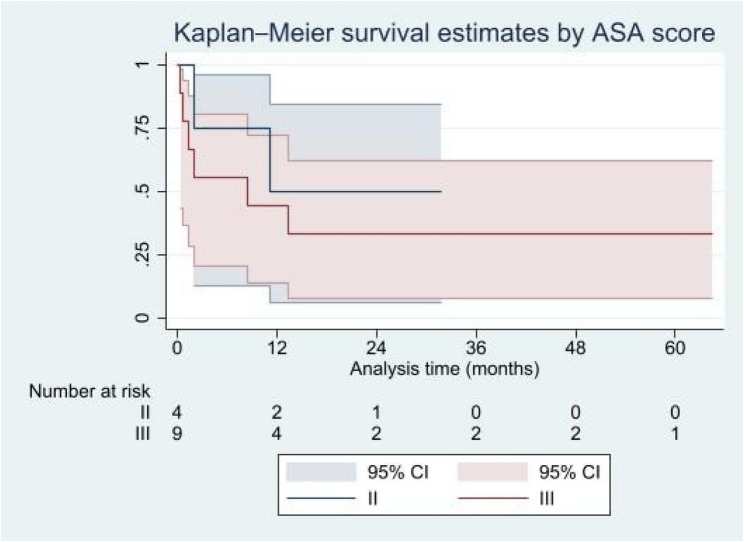
Kaplan‒Meier curves according to the ASA classification and the association with the occurrence of major complications. A positive trend is observed between a higher ASA grade and a higher incidence of major complications

Finally, when patients with different ASA classifications were compared in terms of the presence of major complications, no statistically significant differences were found (log-rank test, χ² = 0.49; *p* = 0.48), as visualized by Kaplan–Meier analysis (Fig. 4). However, patients with higher ASA grades tended to have a higher frequency of complications, suggesting a possible relationship between a worse general condition and an increased risk of adverse events.

## Discussion

The foreseeable increase in prosthetic revision surgeries in the coming years will result in new intraoperative challenges [2, 3]. Bone loss in patients with multiple revision surgeries of the femoral component, especially after knee arthroplasty, inter-/periprosthetic fractures, and persistent joint infections with little bone remnant are challenging conditions for the orthopedic surgery team, since these bone defects, when they affect the femoral isthmus, compromise the fixation and survival of porous diaphyseal anchoring stems when used through conventional methods [8, 17].

In this context, TFR has emerged as a valid alternative for patients requiring such complex prosthetic reconstructions of the hip or knee. Although the initial indications were oncological, its use in nontumor contexts has been increasing in recent years [7, 18, 19].

Several systematic reviews and articles have evaluated the use of TFR as well as its clinical and functional outcomes. Most of these studies, similar to ours, include a small sample size and limited follow-up in the short and medium term (Table 3). In our series of nononcology cases, the main indications for performing TFR were periprosthetic fractures with massive bone loss, aseptic prosthetic loosening and periprosthetic infections, indications that are consistent with those reported in the literature [4, 20, 21]. These conditions represent a significant clinical challenge, since they are usually associated with multiple previous surgeries with deterioration of soft tissues and low bone reserve.

**Table 3:** Summary of the included articles

Article	Patients	Previous surgeries	Follow-up	Indications for total femur replacement	Complications	Functional outcomes
Berend et al. (2004) [25]	59	Hip: mean 3.3 (range 0–15) Knee: mean 1.9	Mean 4.8 years (range 1–13)	Periprosthetic infections Periprostheticf fracture	Infection Dislocation	Harris hip scores (HHS) improved from 40.1 (range, 15–76 of a possible 100) before surgery to 71 (range, 40–88) after surgery. Knee Society score (KSS) improved from 52 (KSS, range, 30–63 of a possible 100) preoperatively to an average of 79 (KSS, range, 33–100).
Friesecke et al. (2005) [5]	100	Hip: mean 3.2 (range 0–12) Knee: mean 0.5 (range 0–11).	Mean 59 months (range 1–138)	Periprosthetic/interprosthetic fracture Aseptic loosening	Periprosthetic infection Dislocation	Preop Enneking Knee score: 2.09 Postop Enneking Knee score: 3.29 Preop Enneking Hip score: 1.25 Postop Enneking Hip score: 3.29
Peters et al. (2006) [19]	22	—	Mean 36 months (range 24–74)	Periprostheticf fracture Aseptic loosening	Periprosthetic infection Dislocation	Postop HHS (revision TKA stem to existing THA stem group): 72 Postop KSS (revision TKA stem to existing THA stem group): 79 Postop HHS (new TKA Stem to new THA stem group): 58 Postop KSS (new TKA stem to new THA stem group): 82
Hoell et al. (2011) [18]	27	- Intramedullary femur replacement: Hip: mean 3.2 (range 1 to 7) Knee: mean 1.5 (range 0 to 6) - Total femoral replacement Hip: mean 3.5 (range 0 to 10) Knee: mean 1.8 (range 0 to 7)	Mean 31.3 months (range 6 to 90)	Periprosthetic infection Aseptic loosening	Complications 37%: Periprosthetic infection Wound healing problem Dislocation.	Postop MSTS (IMFR group): 65 Postop MSTS (TTFR group): 69 Postop HHS (IMFR group): 58 Postop HHS (TTFR group): 61 Postop KSS (IMFR Group): 73 Postop KSS (TTFR group): 77
Amanatullah et al. (2014) [23]	20	—	Mean 73 ± 49 months	Periprosthetic infection Periprosthetic fracture	Infection Dislocation	Preoperative HHS: 30.2 Postoperative HHS: 65.3
Toepfer et al. (2016) [17]	18	Multiple operations (range: 1–8)	Mean 80 months (range, 28–132)	Periprosthetic fracture Aseptic loosening	Type I (soft tissue failure) TypeIV (infection) according to Henderson et al.	HHS (periprosthetic fracture group) at last follow-up: 43 OKS (periprosthetic fracture group) at last follow-up: 14 HHS (aseptic loosening group) at last follow-up: 38 OKS (aseptic loosening group) at last follow-up: 17 SF-12 physical subdomain (both groups) at last follow-up: 27 SF-12 mental subdomain (both groups) at last follow-up: 38
Putman et al. (2019) [6]	29	Mean 3.6 (range 1–10) Hip 3.6 ± 1.8 (max 5) Knee 4.5 ± 2.4 (max 10)	Mean 6 ± 4.5 years	Aseptic loosening Periprosthetic fracture	Surgical site infection Hematoma	—
Graulich et al.(2019) [20]	22	—	Mean 18 months (+/− 21 months)	Periprosthetic infections Periprostheticf fracture	Complications 54%: Periprosthetic infections Dislocation	MSTS score at follow-up: 68
Christ et al. (2020) [22]	16	Mean 4.6 (range 2–9)	Mean 3.9 ± 3.1 years	Periprosthetic fracture Aseptic loosening	Periprosthetic infection	—
Murray et al. (2023) [24]	38	Median 3 (IQR 3 to 5)	Mean 10 years (range, 0–26)	Periprosthetic infections Periprostheticf fracture	Dislocation Periprosthetic infection	—
Ribera et al. (2023) [4]	16	Mean 3 (range 1–7)	Mean 5 years (range 1–9)	Periprosthetic infection. Aseptic loosening	Complications 50%: Seromas Dislocation	HHS mean improved from 26.5 ± 6.8 to 69.5 ± 13.5 Mean MSTS score: 68.1%

Despite obtaining acceptable functional results, the complication rates were high. Periprosthetic infections are currently the main cause of implant failure, with rates ranging between 20% and 35% [19–22]. In our cohort, these complications were also the most frequent, at 38.46%. We believe that this high rate of septic complication is related to the high number of previous surgeries and, consequently, the probable deterioration of soft tissues, as well as the advanced age and comorbidities of the patients.

Dislocation is another important complication, although in our series, we did not record any such cases. We believe that the predominant use of “push-through” rods, which allow maintenance of the greater trochanter with its gluteal insertion, and soft tissue reconstruction techniques contributed to the lack of this type of complication as of the time of writing. The literature supports the importance of reconstruction of the abductor apparatus and soft tissues to reduce the risk of dislocation and reports a relatively low incidence of dislocations associated with “push-through” stems [9]. In contrast, the cohort study published by Hoell et al. indicates a relatively high rate of complications in patients who underwent TFR with this type of implant, especially with regard to both dislocation and periprosthetic infection. The authors attributed the high incidence of infections to the possible compromise of blood circulation due to the preservation of a previously damaged femoral shaft, whereas with most tumor-type prostheses, the patient undergoes radical debridement, and the replacement femur incorporates silver coating with antimicrobial effects. In terms of the rate of dislocations, these differences can be explained by the more frequent use of double-mobility cups in tumor-type prostheses [18]. Other studies have supported the use of constrained or double-mobility cups to reduce and resolve dislocations [9].

A complication observed in our first few patients was fracture of the greater trochanter when a straight rod was introduced over the anatomical curvature of the femur. This is why we always perform an extended trochanteric osteotomy to avoid fractures in the proximal third of the femur, as explained in the surgical technique section.

Another critical aspect is the rate of surgical revision. The percentages reported in the literature vary widely depending on the age and morbidity of the patients, although they are consistently high. However, lower revision rates have been reported in patients treated with “push-through” TFR. In published series, the revision rates ranged between 8.3% and 66.7% for tumor-type TFR and between 20% and 22.7% for patients treated with “push-through” stems [9, 18, 21, 23]. In our series, more than 50% of patients required some type of reintervention in the short or medium term, mainly because of periprosthetic infection, complications related to the surgical wound and one patient who experienced malrotation. Although 84.62% of the patients in our series (11 of 13) received a push-through stem, the revision rates were similar to or even higher than those described in the literature for patients with similar demographic characteristics and numbers of previous surgical interventions [17, 21, 23, 24].

The functional results after TFR in nononcology patients show variability among the different published studies; however, they are considered generally acceptable in the context of the functional demands of the patients, the magnitude of the procedure and the available alternatives [9, 17, 20, 22, 23]. In our cohort, the scores on the PROM questionnaires are lower than those reported by Clement et al. and Hoell et al. but are similar to those described by Toepfer et al. and Graulich et al. in nononcology patients, indicating at least partial agreement with the literature. More specifically, 92.3% of our patients (12 of 13) achieved functional assisted ambulation using crutches or a walker, with moderate overall scores on the MSTS (9.92 ± 3.52). In their systematic review of seven studies, Liu et al. reported that 70% of patients were able to walk, most with some type of assistance [9]. Similarly, Murray et al. reported that 78% of patients achieved assisted ambulation during the postoperative period [24]. With respect to the scores on the MSTS, our results are comparable to those of Toepfer et al., whose nononcological series showed mean values of 9–10 points and confirms that although the functional potential after TFR is limited, this intervention allows preservation of the limb and the achievement of an acceptable level of independence.

Although numerous studies use questionnaires specific to the hip or knee, in our study, we prioritized assessments of generic PROMs of quality of life and functional independence. We believe this aspect is of particular importance. This decision was made in part because patients started from extreme situations, where the main objective was to regain independence and the ability to walk rather than to optimize joint mobility or correct deformities. In addition, the TFR technique includes the replacement of a previously nonpathological joint. The absence of preoperative PROMs constitutes a methodological limitation; however, most TFRs were performed in the context of acute complications. In fact, eight of the thirteen patients in the series had femur fractures; thus, for these patients, the administration of a preoperative functional questionnaire was meaningless.

In terms of pain control, our results revealed an average VAS score of 3.5/10, which aligns with that reported in previous studies. Although some series have reported slightly lower values, the literature agrees on the persistence of a certain degree of residual pain. Nevertheless, the majority of patients in our analysis reported that the pain was controllable and compatible with an acceptable quality of life, which suggests that the presence of persistent discomfort does not necessarily compromise overall satisfaction and reinforces the validity of the TFR as a strategy for managing femoral bone loss [5, 22, 25].

Previous studies have indicated that the number of previous surgeries, the age of the patient and the indications for TFR are associated with subsequent functional evolution after the intervention and thus serve as prognostic factors for patient outcomes [17, 21]. However, in our series, a statistically significant correlation between these potential prognostic factors and functional outcomes was not identified. Although our literature review identified studies that do describe such associations, we also found studies whose findings coincide with ours [5, 17, 24]. This discrepancy in the literature is likely a consequence of the heterogeneity of the indications as well as the small sample size of many of the available studies, which limits the statistical power to identify consistent prognostic factors.

In several studies, TFR has been proposed as the only viable alternative to amputation or joint resection without reconstruction [22, 24]. However, for most of our patients, this dichotomy was not considered the main driver in decision-making. This is explained by the fact that, in our series, the most frequent indication for TFR was the presence of peri-/interprosthetic fractures, which generally allows the possibility of preserving the limb.

Our study demonstrates limitations inherent to its retrospective nature and small sample size, as well as methodological limitations, such as the absence of PROM information prior to the TFR and the lack of a comparison group. In this sense, statistical analyses were not intended to establish definitive associations, but rather to explore potential trends that may be clinically relevant in this uncommon and challenging scenario. However, our results seem to be comparable to those published in the literature, which has reported similar limitations. Despite this, in elderly patients with low functional demand and multiple surgical failures, TFR represents a therapeutic option that offers improved patient quality of life and independence. As proposed by Liu et al., in nononcology patients, TFR should be considered a rescue option rather than a first-line technique. Collectively, these findings emphasize the need for a careful evaluation of the supposed indication as well as a highly specialized and multidisciplinary surgical approach.

This work provides valuable clinical evidence in a field where the literature is still scarce and therapeutic decisions are often based on small series and the individual experience of each center. Our cohort specifically included nononcology patients who underwent TFRs, with systematic follow-up and a detailed analysis of functional results, pain control and complications. These data contribute to more precisely defining the therapeutic role of TFR and improving the ability of surgeons to select appropriate patients and establish realistic expectations in complex scenarios where surgical alternatives are extremely limited. Overall, we believe that the findings complement the existing evidence and provide relevant information applicable to clinical practice, reinforcing the need for multicenter prospective studies with larger samples and longer follow-ups to confirm these findings, define the prognostic factors and patient selection criteria more accurately, reduce the rate of associated complications and establish this reconstructive strategy more solidly.

## Conclusions

In patients with severe femoral bone defects and femoral isthmus compromise, TFR is a viable therapeutic choice when there are no other surgical options. Despite the high rate of complications described in this study, this technique allows recovery of walking ability and some degree of functional independence as well as adequate pain control, offering a reasonable reconstructive solution in these complex scenarios.

## Data Availability

The datasets used and/or analyzed during the current study are available from the corresponding author on reasonable request.

## Abbreviations

CPI: chronic periprosthetic infection
KW: Kruskal‒Wallis test
MSTS: Musculoskeletal Tumor Society score
TFR: Total femoral replacement
PAI: acute periprosthetic infection
PROMs: Patient-reported outcome measures
SF-12: 12-item short form questionnaire
WOMAC: Western Ontario and McMaster Universities Osteoarthritis Index
VAS: Visual Analog Scale

## References

[1] Sloan M, Premkumar A, Sheth NP, the U.S. Projected Volume of Primary Total Joint Arthroplasty in, 2014 to 2030. JBJS. 2018;100(17). Disponible en: https://journals.lww.com/jbjsjournal/fulltext/2018/09050/projected_volume_of_primary_total_joint.3.aspx. 10.2106/JBJS.17.01617 30180053

[2] Kurtz S, Ong K, Lau E, Mowat F, Halpern M. Projections of Primary and Revision Hip and Knee Arthroplasty in the United States from 2005 to 2030. JBJS. 2007;89(4). Disponible en: https://journals.lww.com/jbjsjournal/fulltext/2007/04000/projections_of_primary_and_revision_hip_and_knee.12.aspx. 10.2106/JBJS.F.00222 17403800

[3] Shichman I, Askew N, Habibi A, Nherera L, Macaulay W, Seyler T, et al. Projections and Epidemiology of Revision Hip and Knee Arthroplasty in the United States to 2040–2060. Arthroplasty Today junio de. 2023;21:101152. 10.1016/j.artd.2023.101152 PMC10244911 37293373

[4] Ribera J, Payo-Ollero J, Serrano-Toledano D, Del Río-Arteaga M, Montilla FJ, Muela R. Megaprosthesis use in Paprosky III/IV femoral defects in non-oncological patients: analysis of survival, clinical, and functional outcomes after an average follow-up of five years. Eur J Orthop Surg Traumatol 25 de noviembre de. 2023;34(2):1183–92. 10.1007/s00590-023-03783-9 38006463

[5] Friesecke C, Plutat J, Block A. Revision Arthroplasty with Use of a Total Femur Prosthesis. JBJS. 2005;87-A(12). 10.2106/JBJS.D.02770 16322619

[6] Putman S, Migaud H, Saragaglia D, Jenny JY, Dujardin F, Hue AG, et al. Total femur replacement in non-oncologic indications: Functional and radiological outcomes from a French survey with a mean 6 years’ follow-up. Orthop Traumatol Surg Res junio de. 2019;105(4):591–8. 10.1016/j.otsr.2018.12.013 31027981

[7] Wilk B, Rojek M, Gugulska J, Kasprzak P, Wrześniak Z, Pulik Ł, et al. Total femur replacement, indications for the procedure and its complications: a systematic review. Arch Orthop Trauma Surg 29 de abril de. 2025;145(1):278. 10.1007/s00402-025-05887-9 PMC12041181 40301142

[8] DeRogatis MJ, Issack PS. Total Femoral Replacement as a Salvage Operation for the Treatment of Massive Femoral Bone Loss During Revision Total Hip Arthroplasty. JBJS Rev mayo de. 2018;6(5):e9–9. 10.2106/JBJS.RVW.17.00195 29847442

[9] Liu CM, Ehlers CB, Berger GK, Ball ST, Chiarappa FE. Total femur replacement in revision arthroplasty for non-oncologic patients: a systematic review. Eur J Orthop Surg Traumatol 12 de marzo de. 2025;35(1):112. 10.1007/s00590-025-04226-3 40074982

[10] Aribindi R, Barba M, Solomon MI, Arp P, Paprosky W. Bypass fixation. Orthop Clin North Am. 1998;29(2):319–29. 9553577 10.1016/s0030-5898(05)70330-8

[11] Della Valle CJ, Paprosky WG. Classification and an Algorithmic Approach to the Reconstruction of Femoral Deficiency in Revision Total Hip Arthroplasty. J Bone Jt Surg-Am Vol. 2003;85:1–6. 10.2106/00004623-200300004-00001 14652388

[12] Ware JE. Kosinski M, Keller SD. A 12-Item Short-Form Health Survey: Construction of Scales and Preliminary Tests of Reliability and Validity. Med Care. 1996;34(3). Disponible en: https://journals.lww.com/lww-medicalcare/fulltext/1996/03000/a_12_item_short_form_health_survey__construction.3.aspx. 10.1097/00005650-199603000-00003 8628042

[13] Enneking WF, Dunham W, Gebhardt MC, Malawar M, Pritchard DJ. A system for the functional evaluation of reconstructive procedures after surgical treatment of tumors of the musculoskeletal system. Clin Orthop enero de. 1993;286:241–6. 8425352

[14] Bellamy N, Buchanan WW, Goldsmith CH, Campbell J. Validation study of WOMAC: a health status instrument for measuring clinically important patient relevant outcomes to antirheumatic drug therapy in patients with osteoarthritis of the hip or knee. J Rheumatol 15 de diciembre de. 1988;12:1833–40. 3068365

[15] Jensen MP, Karoly P, Braver S. The measurement of clinical pain intensity: a comparison of six methods. PAIN [Internet]. 1986;27(1). Disponible en: https://journals.lww.com/pain/fulltext/1986/10000/the_measurement_of_clinical_pain_intensity__a.10.aspx. 10.1016/0304-3959(86)90228-9 3785962

[16] McNally M, Sousa R, Wouthuyzen-Bakker M, Chen AF, Soriano A, Vogely HC, et al. Infographic: The EBJIS definition of periprosthetic joint infection: a practical guide for clinicians. Bone Jt J enero de. 2021;103–B(1):16–7. 10.1302/0301-620X.103B1.BJJ-2020-2417 PMC7954145 33380197

[17] Toepfer A, Harrasser N, Petzschner I, Pohlig F, Lenze U, Gerdesmeyer L, et al. Short- to long-term follow-up of total femoral replacement in non-oncologic patients. BMC Musculoskelet Disord diciembre de. 2016;17(1):498. 10.1186/s12891-016-1355-6 PMC5154048 27955655

[18] Hoell S, Butschek S, Gosheger G, Dedy N, Dieckmann R, Henrichs M, et al. Intramedullary and total femur replacement in revision arthroplasty as a last limb-saving option: Is there any benefit from the less invasive intramedullary replacement? J Bone Joint Surg Br noviembre de. 2011;93–B(11):1545–9. 10.1302/0301-620X.93B11.27309 22058309

[19] Peters CL, Hickman JM, Erickson J, Lombardi AV, Berend KR, Mallory TH. Intramedullary Total Femoral Replacement for Salvage of the Compromised Femur Associated With Hip and Knee Arthroplasty. J Arthroplasty enero de. 2006;21(1):53–8. 10.1016/j.arth.2004.12.061 16446185

[20] Graulich T, Steimer D, Zhang D, Omar M, Weber-Spickschen S, Krettek C, et al. High complication and revision rates after total femoral replacement: a retrospective single center analysis of indication, function, and complication. Arch Orthop Trauma Surg julio de. 2019;139(7):913–20. 10.1007/s00402-019-03130-w 30687872

[21] Ramanathan D. Current concepts in total femoral replacement. World J Orthop. 2015;6(11):919. 26716087 10.5312/wjo.v6.i11.919 PMC4686438

[22] Christ AB, Mendez L, Gausden EB, Blevins JL, Bostrom MP, Sculco PK. Outcomes and complications following non-oncologic total femoral replacement. HIP Int noviembre de. 2020;30(6):725–30. 10.1177/1120700019864867 31317783

[23] Amanatullah DF, Trousdale RT, Hanssen AD, Lewallen DG, Taunton MJ. Non-Oncologic Total Femoral Arthroplasty: Retrospective Review. J Arthroplasty octubre de. 2014;29(10):2013–5. 10.1016/j.arth.2014.05.012 25041874

[24] Murray J, Jeyapalan R, Davies M, Sheehan C, Petrie M, Harrison T. Total femoral arthroplasty for non-oncological indications. Bone Jt J. 2023;105–B(8):888–94. 10.1302/0301-620X.105B8.BJJ-2022-1372.R1 37524348

[25] Berend KR, Lombardi AV, Mallory TH, Adams JB, Dodds KL. Total Femoral Arthroplasty for Salvage of End-stage Prosthetic Disease: Clin Orthop. octubre de 2004;427:16 –70. 10.1097/01.blo.0000142351.88039.e8 15552153

